# Supplementary material for: Resolving the full spectrum of human genome variation using Linked-Reads
Source: Genome Res. 2019 Apr;29(4):635–45. doi: 10.1101/gr.234443.118 (PMC6442396; doi:10.1101/gr.234443.118)
Supplement: Supplemental Material [file supp_gr.234443.118_Supplemental_longranger-2.2.zip › longranger-2.2.tar.bz2!/lr2.2-public/lib/stan/cmdstan/stan/doc/api/html/structstan_1_1size__of__helper_3_01_t_00_01true_01_4.html]

Stan: stan::size\_of\_helper< T, true > Struct Template Reference


|  |  |
| --- | --- |
|  | Stan  2.5.0  probability, sampling & optimization |


- Main Page
- Related Pages
- Namespaces
- Classes
- Files

- Class List
- Class Index
- Class Hierarchy
- Class Members

All Classes Namespaces Files Functions Variables Typedefs Enumerations Enumerator Friends Macros Pages

- stan
- size\_of\_helper< T, true >

Static Public Member Functions |
List of all members

stan::size\_of\_helper< T, true > Struct Template Reference

`#include <traits.hpp>`

|  |  |
| --- | --- |
| Static Public Member Functions | |
| static size\_t | size\_of (const T &x) |
|  | |

## Detailed Description

### template<typename T> struct stan::size\_of\_helper< T, true >

Definition at line 179 of file traits.hpp.

## Member Function Documentation

template<typename T >

|  |  |  |  |  |  |
| --- | --- | --- | --- | --- | --- |
| static size\_t stan::size\_of\_helper< T, true >::size\_of | ( | const T & | *x* | ) |  |

inlinestatic

Definition at line 180 of file traits.hpp.

---

The documentation for this struct was generated from the following file:

- src/stan/meta/traits.hpp


---

[ Stan Home Page ]

*© 2011–2014,
Stan Development Team.*
